# Auditory Brain Stem Responses in the C57BL/6J Fragile X Syndrome-Knockout Mouse Model

**DOI:** 10.3389/fnint.2021.803483

**Published:** 2022-01-17

**Authors:** Amita Chawla, Elizabeth A. McCullagh

**Affiliations:** Department of Integrative Biology, Oklahoma State University, Stillwater, OK, United States

**Keywords:** auditory brainstem response (ABR), Fragile X Syndrome, binaural hearing, sex differences, mouse model

## Abstract

Sensory hypersensitivity, especially in the auditory system, is a common symptom in Fragile X syndrome (FXS), the most common monogenic form of intellectual disability. However, linking phenotypes across genetic background strains of mouse models has been a challenge and could underly some of the issues with translatability of drug studies to the human condition. This study is the first to characterize the auditory brain stem response (ABR), a minimally invasive physiological readout of early auditory processing that is also used in humans, in a commonly used mouse background strain model of FXS, C57BL/6J. We measured morphological features of pinna and head and used ABR to measure the hearing range, and monaural and binaural auditory responses in hemizygous males, homozygous females, and heterozygous females compared with those in wild-type mice. Consistent with previous study, we showed no difference in morphological parameters across genotypes or sexes. There was no significant difference in hearing range between the sexes or genotypes, however there was a trend towards high frequency hearing loss in male FXS mice. In contrast, female mice with homozygous FXS had a decreased amplitude of wave IV of the monaural ABR, while there was no difference in males for amplitudes and no change in latency of ABR waveforms across sexes and genotypes. Finally, males with FXS had an increased latency of the binaural interaction component (BIC) at 0 interaural timing difference compared with that in wild-type males. These findings further clarify auditory brain stem processing in FXS by adding more information across genetic background strains allowing for a better understanding of shared phenotypes.

## Introduction

Fragile X syndrome (FXS) is the most common monogenic form of autism spectrum disorder (ASD) and shares many attributes of ASDs, including auditory hypersensitivity and other sensory disruptions (Abbeduto and Hagerman, 1997; Chen and Toth, 2001; Hagerman and Hagerman, 2002; Arnett et al., 2014). FXS is a tractable genetic model for ASD with several commercially available models, including the rat and mouse (The Dutch-Belgian Fragile X Consorthium et al., 1994; Till et al., 2015; Tian et al., 2017). Despite the common use of these models to study the FXS, phenotypes are not always shared between species and background strains, particularly for sensory processing. As a result, drug therapies have struggled to rescue the human disorder (Dahlhaus, 2018). One of the most common symptoms described in people with FXS and autism spectrum disorder (ASD) is auditory hypersensitivity (Ethridge et al., 2017; Stefanelli et al., 2020). Clinically, auditory phenotypes present as reduced auditory attention, impaired habituation to auditory stimuli, reduced prepulse inhibition of acoustic startle, and overall hypersensitivity to auditory conditions (reviewed in Sinclair et al., 2017; Rais et al., 2018; Razak et al., 2021) that have likely both cortical and subcortical origins. Indeed, much of the research in this area has focused on cortical measures of auditory phenotypes, which receive inputs from lower auditory regions that may also be disrupted but less likely to be measured clinically. The mechanisms that underly auditory alterations are unknown, but likely involve the entirety of the ascending pathway from the periphery to the cortex (reviewed in McCullagh et al., 2020b). A complete characterization of auditory processing from the periphery to cortex across sexes, background strains, and models is needed to fully understand shared phenotypes and circuitry involved in this common symptom.

The auditory brain stem is one brain region in the ascending auditory pathway that has been shown to have anatomical, physiological, and behavioral alterations in mouse models with FXS (Brown et al., 2010; Beebe et al., 2014; Wang et al., 2014, 2015; Rotschafer et al., 2015; Garcia-Pino et al., 2017; McCullagh et al., 2017, 2020a; Rotschafer and Cramer, 2017; Curry et al., 2018; El-Hassar et al., 2019; Lu, 2019) that likely underly or contribute to the overall auditory phenotypes exhibited in both humans and animal models. Much like auditory hypersensitivity in humans, mice exhibit changes to the prepulse inhibition to the acoustic startle response, abnormal EEG activity, and, in the most extreme form, audiogenic seizures when presented with loud sounds (Chen and Toth, 2001; Lovelace et al., 2018, 2020; McCullagh et al., 2020a), making them a potentially suitable model for this sensory phenotype. The auditory brain stem is the first site of binaural processing of sound location in the brain using interaural timing and level differences (i.e., ITD and ILD, respectively) to compute sound source locations (Grothe et al., 2010). This brain area is also involved in separating spatial channels allowing for complex listening environments. Disruptions in this spatial separation and binaural processing could lead to auditory hypersensitivity due to the inability to separate sound sources (Bronkhorst, 2015). One measure of auditory brain stem physiology, and binaural hearing, that can be directly translated between animal models and humans is the auditory brain stem response (ABR) (Laumen et al., 2016).

The ABR is a minimally invasive physiological measure that allows for a simultaneous assessment of sound processing across multiple brain stem nuclei, as each wave of the ABR directly corresponds to distinct areas of the ascending auditory brain stem pathway. These features make the ABR an attractive translational tool. Indeed, recent evidence suggests that ABR measurements are an early indicator of auditory dysfunction in ASD (Santos et al., 2017). ABRs can also be used to assess binaural hearing, which is essential for sound localization and hearing in noisy environments and often impaired in ASD (Visser et al., 2013). Monoaural ABRs can be recorded by stimulating each ear separately, and binaural responses can be generated by stimulating both ears simultaneously. The sum of the two monaural (i.e., left and right) responses should equal the binaural (i.e., both ear) responses since the recruited neural activity from each ear should be double when stimulated simultaneously. However, this is not the case, there is a difference that arises when the summed monoaural responses are subtracted from the binaural response, called the binaural interaction component (BIC). The BIC is thought to be a direct measure of binaural processing ability in humans and animals that requires the precise balance of excitatory and inhibitory drive in brain stem sound localization circuits (Laumen et al., 2016).

In this study, we reported on the hearing ability, using the ABR and morphological craniofacial and pinna features, of the most common mouse model with FXS, C57BL/6J across the sexes and females heterozygous for the Fmr1 mutation. We hypothesized that there may be sex differences in ABRs independent of the FXS genotype, but that in addition, FXS animals are likely to have alterations in peak amplitude or latency of ABRs and impaired high-frequency hearing compared with wild-type consistent with work in other mouse strains with FXS (Kim et al., 2013; Rotschafer et al., 2015; El-Hassar et al., 2019). Establishing core auditory phenotypes across the sexes and different mouse strains is key to creating a toolbox of techniques that may translate to human FXS both to validate the utility of animal models to human conditions but also add to potential measures for the efficacy of the drug or other treatment options.

## Materials and Methods

All experiments complied with all applicable laws, National Institutes of Health guidelines, and were approved by the Oklahoma State University IACUC.

### Animals

Experiments were conducted in C57BL/6J (stock #000664, B6) wild-type background, hemizygous male, homozygous male and female, or heterozygous female *Fmr1* mutant mice (B6.129P2-*Fmr1*^TM 1*Cgr*^/J stock #003025, Fmr1 or Fmr1 het, respectively) obtained from the Jackson Laboratory and bred at Oklahoma State University (Bar Harbor, ME, United States) (The Dutch-Belgian Fragile X Consorthium et al., 1994). Animals were generated for these experiments from stocks by both mixed and single genotype mating allowing for the creation of heterozygotes and some littermate controls, as well as maintenance of breeding lines. There was no significant main effect of litter (i.e., mixed or single genotype) for any of the experiments. Sex was treated as a biological variable, and differences between the sexes, when present, are noted in the results. The numbers of animals for each experiment used are listed in the figure legends and range from 6–10 animals per sex and genotype. Animals ranged in age from 62–120 days (i.e., average ages per genotype 89 ± 4 days B6, 101 ± 3 days Fmr1, and 97 ± 4 days Fmr1 het).

### Morphological Measures

**FIGURE 1:**
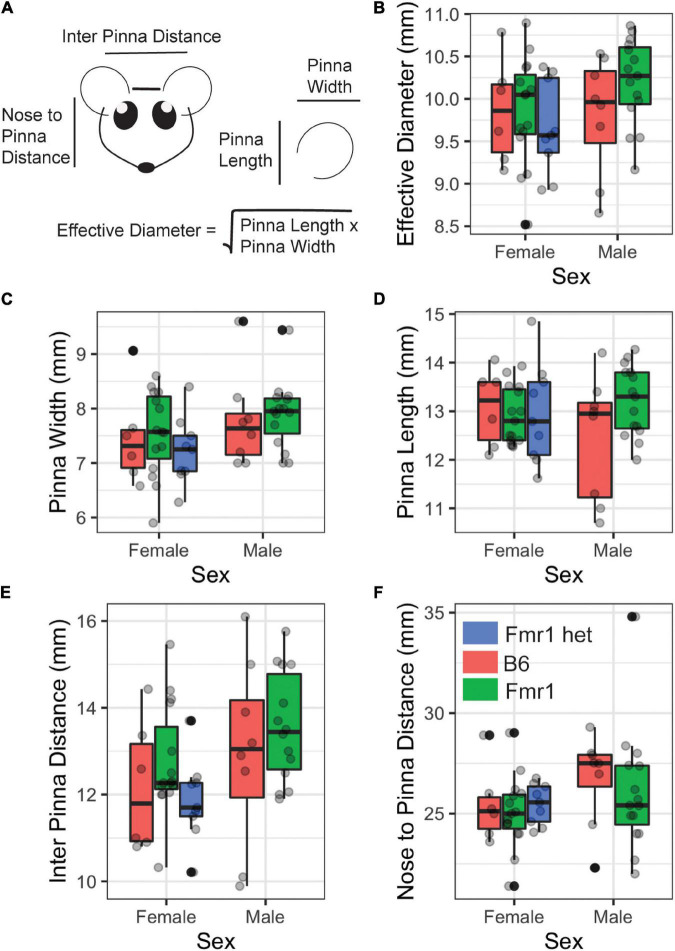
Morphological features of Fragile X syndrome (FXS) mice. Pinna and head features **(A)** were measured between the sexes (*x*-axis) and genotypes (purple = B6, teal = Fmr1, and yellow = Fmr1 het). There was no difference between the sexes or genotypes for any of the measures [effective diameter **(B)**, pinna width **(C)**, pinna length **(D)**, interpinna length **(E)**, or nose to pinna length **(F)**]. Data represent 6 B6, 15 Fmr1, and 9 Fmr1 het females and 8 B6 and 15 Fmr1 males.

Features of animal’s head, pinna, and body mass (weight) were measured for each genotype using 6 Inch Stainless Steel Electronic Vernier Calipers (DIGI-Science Accumatic digital caliper Gyros Precision Tools Monsey, NY, United States) and an electronic scale. The distance between the two pinnae (i.e., interpinna distance), distance from the nose to the middle of the pinna (i.e., nose to pinna distance), and pinna width and length were measured (Figure 1A). The effective diameter was calculated as the square root of pinna length times pinna width (Anbuhl et al., 2017).

### Auditory Brain Stem Responses

Auditory brain stem response recordings were performed using similar methods from previously published study (Benichoux et al., 2018; McCullagh et al., 2020a; New et al., 2021). Animals were anesthetized using two mixtures of ketamine-xylazine 60 mg/kg ketamine and 10 mg/kg xylazine for initial induction followed by maintenance doses of 25 mg/kg ketamine and 12 mg/kg xylazine. Once anesthesia was confirmed by lack of a toe-pinch reflex, animals were transferred to a small sound attenuating chamber (Noise Barriers Lake Forest, IL, United States), and the body temperature was maintained using a water-pump heating pad. Subdermal needle electrodes were placed under the skin between the ears (i.e., apex), directly behind the apex in the nape (i.e., reference), and in the back leg for ground. This montage has been shown to be particularly effective in generating the BIC (Levine, 1981; Laumen et al., 2016). Evoked potentials from subdermal needle electrodes were acquired and amplified using Tucker-Davis Technologies (TDT, Alachua, FL, United States) RA4LI head stage and a TDT RA16PA preamplifier. Further amplification was provided by a TDT Multi I/O processor RZ5 connected to a PC with custom Python software for data recording. Data were averaged across 500–1,000 repetitions per condition and processed using a second-order 50–3,000 Hz filter over 12 ms of recording time.

Sound stimuli (refer below for varying types) were presented to the animal through TDT EC-1 electrostatic speakers (frequencies 32–46 kHz) or TDT MF-1 multifield speakers (frequencies 1–24 kHz and broadband clicks) coupled through custom earpieces fitted with Etymotic ER-7C probe microphones (Etymotic Research Inc., Elk Grove Village, IL, United States) for the in-ear calibration (Beutelmann et al., 2015). Sounds were generated using a TDT RP2.1 Real-Time processor controlled by the custom Python code at a sampling rate of 97656.25 Hz. Sounds were presented at an interstimulus interval of 30 ms with a standard deviation of 5 ms (Laumen et al., 2016). An additional rejection threshold was set to eliminate high-amplitude heart rate responses from average traces and improve the signal-to-noise ratio.

#### Audiogram

The hearing range of animals was tested using the threshold for hearing across different frequencies (i.e., 1, 2, 4, 8, 16, 24, 32, 46 kHz) of sound. Threshold was determined using a visual detection method (Brittan-Powell and Dooling, 2004), or the lowest level (dB SPL) a response could be detected. Audiogram stimuli consisted of tone bursts (2 ms ± 1 ms on/off ramps) of varying frequency and intensity.

#### Monaural Auditory Brain Stem Responses

**FIGURE 2:**
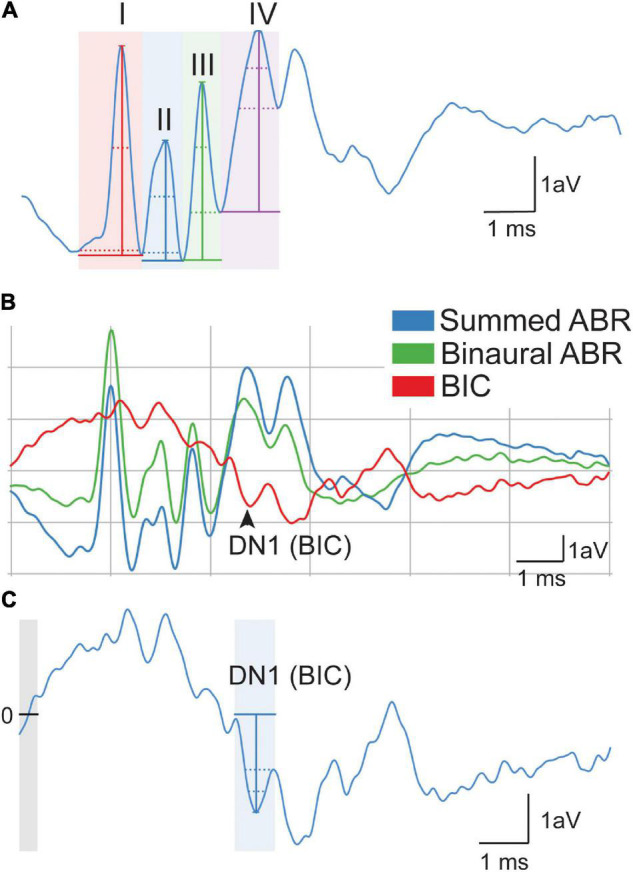
Quantification of auditory brain stem response (ABR) signals. Monaural ABR amplitudes were quantified for each ear as the voltage between the peak of the ABR and trough of the waveform for waves I–IV **(A)**. Latency was calculated as the time when the height of the peak occurred. DN1 or binaural interaction component (BIC) (i.e., red) was calculated as the prominent negative peak corresponding with wave IV of the summed (blue) and binaural (green) **(B)**. BIC is calculated as the summed ABR subtracted from the binaural ABR. The BIC amplitude was calculated as the voltage at the peak of the DN1 waveform to the baseline (0, line, and gray area) of the measurement **(C)**. The scale represents 1 arbitrary voltage (aV) unit (Y) during 1 ms (X).

Broadband click stimuli (i.e., 0.1 ms transient) were presented to each ear independently to generate monaural evoked potentials. Peak amplitude (i.e., the voltage from peak to trough) and latency (i.e., time to peak amplitude) were measured across the four peaks of the ABR waveform at 90 dB SPL (Figure 2A). The trough was considered the lowest point for that wave. Monaural data from the two ears were averaged to determine the monaural amplitude and latency for each animal. Similar to hearing thresholds across frequency, click threshold was determined for each genotype and sex. Click threshold is determined by decreasing the intensity of sound in 5–10 dB SPL steps until ABR waveforms disappear.

#### Binaural Auditory Brain Stem Responses

Broadband click stimuli at 90 dB SPL were also presented to both ears simultaneously to generate a binaural evoked potential. The BIC of the ABR was calculated by subtracting the sum of the two monaural ABRs from the binaural ABR (Laumen et al., 2016; Benichoux et al., 2018) (Figures 2B,C). BIC amplitude and latency were then measured using the custom Python software, with amplitude being relative to the zero baselines of the measurement (Figure 2C, gray area with line). BIC was characterized as the prominent negative DN1 wave corresponding to the fourth wave of the binaural and summed ABR (Figure 2B). To measure ITD computation using the BIC, animals were presented with stimuli that had varying ITDs of ± 2 ms in 0.5 ms steps, and corresponding BIC amplitudes and latencies were calculated like above. This ITD range was chosen to be comparable to other studies in small rodents (Benichoux et al., 2018).

### Analysis of Auditory Brain Stem Response Waveforms

The custom python software was used to analyze evoked potentials for monaural and binaural stimuli (New et al., 2021). To account for fluctuation in the baseline signal of the ABR, raw traces were zeroed to establish a baseline across traces. The software included automatic peak detection with the capability of manual correction or deselection upon visual confirmation.

### Statistical Analyses

**FIGURE 3:**
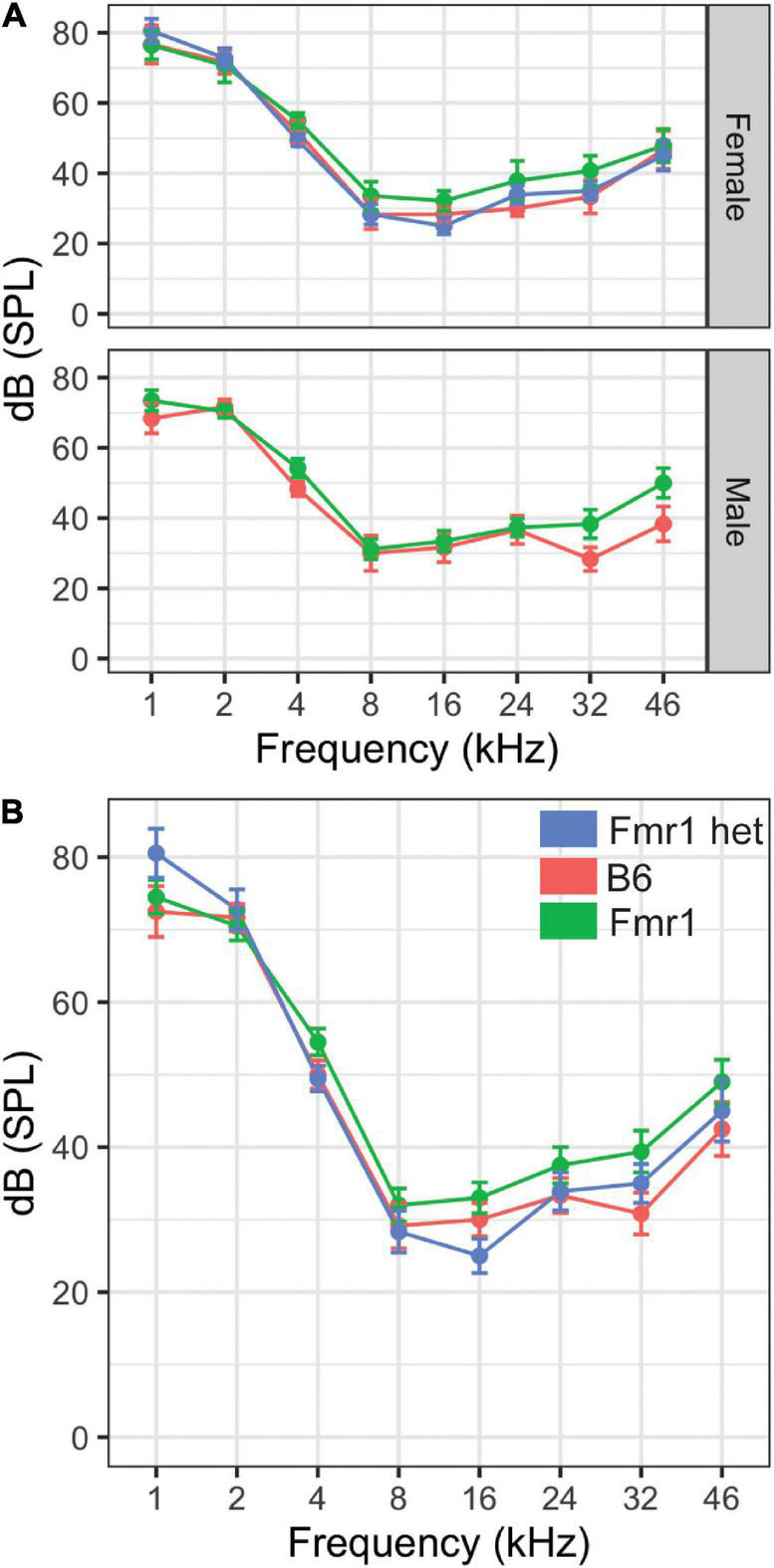
Hearing threshold (dB SPL) was measured across frequencies (1–46 kHz) in male and female mice of all genotypes **(A)**. There were no differences in the hearing range between Fmr1 (green), B6 (red), and Fmr1 het (blue) mice of either sex (top panel **A**). When sexes were combined, there was no significant difference in hearing across frequencies **(B)**. Data represent 6 B6, 7 Fmr1, 9 Fmr1 het females and 6 B6, 11 Fmr1 males.

**FIGURE 4:**
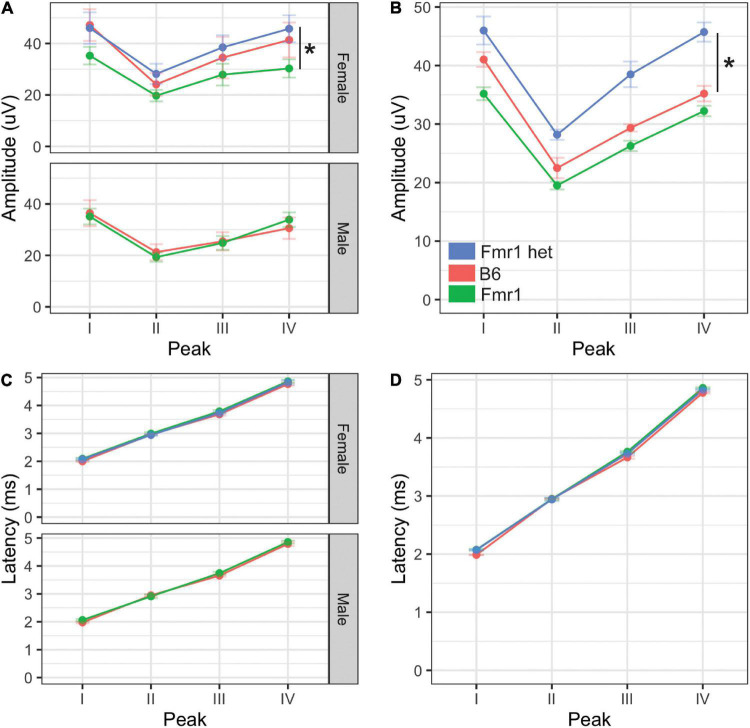
Monaural hearing in mice with FXS. Monaural amplitudes and latencies for peaks I–IV of the ABR were recorded for Fmr1, Fmr1 het, and B6 animals. Peak IV amplitude was significantly lower in Fmr1 mice females compared with Fmr1 het females (**A**, upper). There were no significant differences in amplitudes for males (**A**, lower). When combined, there was a significant difference in Fmr1 het animals compared with Fmr1 **(B)**. There was no difference in latency of peaks I–IV between sexes **(C)** or genotypes **(D)**. *p < 0.05. Data represent 6 B6, 12 Fmr1, and 9 Fmr1 het females and 8 B6 and 14 Fmr1 males.

**FIGURE 5:**
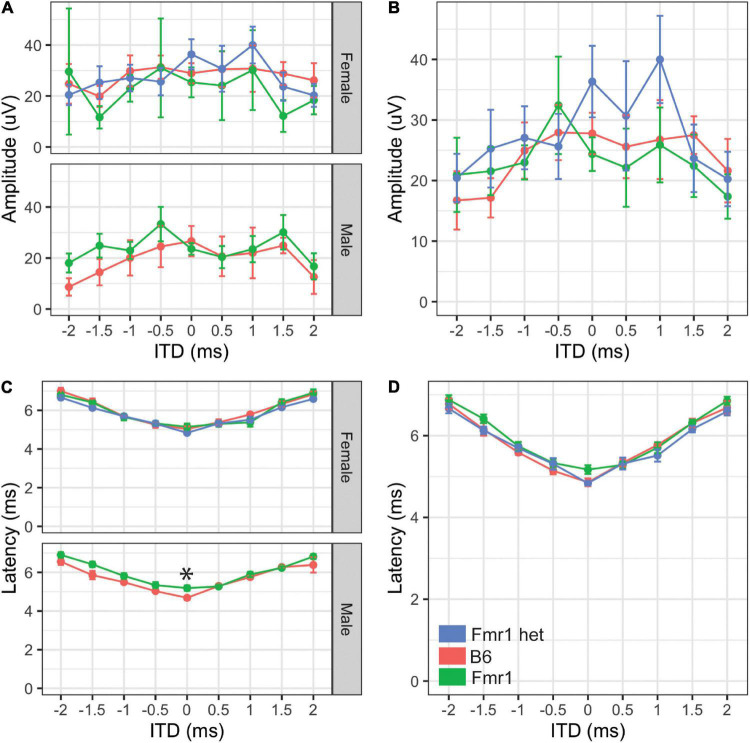
Binaural hearing in Fragile X syndrome (FXS) mice. Binaural amplitudes and latencies for the BIC at ITDs between –2 to + 2 ms in 0.5 ms steps were recorded for Fmr1 (green), Fmr1 het (blue), and B6 (red) animals. No differences in amplitude of the BIC with ITD for females (**A**, upper) or males (**A**, lower). When the sexes were combined, there was no significant difference in amplitude of the BIC with ITD **(B)**. Fmr1 males had significantly longer latency of the BIC at 0 ITD compared with B6 males (C, lower), while there was no difference in latency of female responses (**C**, upper). When the sexes were combined, there was no difference in the BIC latency across ITDs between the genotypes **(D)**. *p < 0.05. Data represent 6 B6, 7 Fmr1, and 9 Fmr1 het females and 6 B6 and 9 Fmr1 males.

Figures were generated using R Studio (R Core Team, 2013), ggplot2 (Wickham, 2016), and Adobe Illustrator (Adobe, San Jose, CA, United States) software. Data points in Figures 3, 4, and 5 represent means, error bars reflect standard error, boxplots in Figure 1 display the median and 25–75th percentiles (or 1st and 3rd quartiles, respectively), the whiskers represent ± 1.5 times the interquartile range. The data that falls outside the range are plotted as individual points. Multivariate data (i.e., monaural peak amplitude and latency, audiogram, and BIC amplitude and latency across ITD) were analyzed using linear mixed effects (lme4) models (Bates et al., 2015) with sex, genotype, litter, and condition (i.e., ITD, frequency, peak) as fixed effects and animal as a random effect. It was expected that there may be differences between the sexes and genotypes; therefore, *a priori*, it was determined that estimated marginal means [emmeans; (Lenth, 2019)] would be used for pairwise comparisons between sexes and genotype. Two-way ANOVAs were performed to compare relationships between morphological features, sex, and genotype with the adjusted Tukey *post hoc* analysis to compare groups. Where values are indicated as statistically significant between the two genotypes, * indicated a *p*-value of <0.05, ^**^p < 0.01, and ^***^p < 0.0001.

## Results

We used both morphological and physiological features to examine hearing differences in a commonly used mouse model with FXS, C57BL/6J across genotypes and sexes. Hearing measurements included the frequency hearing range, monaural hearing ability, and binaural processing using the ABR, while morphological features included pinna and head measurements.

### Morphological Features

People with FXS have altered craniofacial features, including large ears (Loesch et al., 1988). Consistent with our previous work (McCullagh et al., 2020a), we saw no difference between B6, Fmr1, or Fmr1 het animals for pinna attributes (Figure 1C pinna width, Figure 1D pinna length, Figure 1B effective diameter). In addition, pinna characteristics were the same between the sexes independent of genotype (*p* = 0.175 pinna width, *p* = 0.96 pinna length, *p* = 0.267 effective diameter Figures 1B–D). When genotypes were compared within the same sex, there were no differences in weight, but sexes were significantly different independent of genotype (*p* = 0.0023) with females weighing significantly less than males. Similar to the pinna morphology, there was no significant difference in either distance between pinna or distance from the nose to pinna between the genotypes or sexes (Figures 1E,F). These data suggest that mice do not share the same craniofacial changes, at least in the measurements described here, as people with FXS.

### Hearing Range

Our previous study showed that Fmr1 mice have increased thresholds for high-frequency hearing compared with those in B6 at 16 kHz (McCullagh et al., 2020a). However, that study was limited by measuring only three frequencies (i.e., 4, 8, and 16 kHz) and seven mice of each genotype (i.e., combined sexes). Mice hear much higher frequencies than humans (Radziwon et al., 2009); therefore, we wanted to measure whether this high-frequency hearing loss exists across the frequencies in which mice hear in Fmr1 mutants and with a more in-depth sex-specific analysis. Interestingly, there were no differences between genotypes across the frequencies tested (Figure 3). There were no significant differences in hearing range between the sexes. Best frequencies for both genotypes, as indicated by lower threshold, of mice were between 8–46 kHz consistent with specialized high frequency hearing.

### Monaural Hearing

Amplitude and latency of monaural ABRs correspond with the neural activity across the ascending auditory pathway, with each wave representing different brain areas involved in the auditory processing (Alvarado et al., 2012). Other studies have shown both latency and amplitude alterations in the FVB mouse strain of Fmr1 mutation (Kim et al., 2013; Rotschafer et al., 2015; El-Hassar et al., 2019). We measured ABR responses of Fmr1 mutants to monaural click stimuli compared with B6 mutant mice to determine if they have a similar ABR phenotype to the FVB strain. We saw no differences in overall click threshold for either genotype or sex (*p* = 0.102 genotype and *p* = 0.47 for sex). The amplitude of monaural responses was significantly lower for wave IV of the ABR in Fmr1 females compared with Fmr1 het females (Figure 4A upper). Indeed, Fmr1 het female amplitudes were closer to B6 than Fmr1 females, though Fmr1 females were not significantly different from B6. In contrast, Fmr1 male amplitudes for waves I–IV were not different from B6 (Figure 4A lower). When sexes were combined, Fmr1 het females had significantly higher amplitudes than B6 and were close to being significantly higher than Fmr1 mice (*p* = 0.0593). Consistent with sex driving the differences in genotype, peak amplitudes varied between the sexes. Female B6 mice had significantly higher amplitude peaks I and IV compared with B6 males (*p* = 0.0295 peak I and *p* = 0.0289 peak IV). In contrast, there were no sex differences between male and female Fmr1 mice, suggesting a more male-like phenotype (i.e., independent of genotype) in homozygous Fmr1 females. There were no differences between the sexes or genotypes in latency of monaural peaks (Figures 4C,D).

### Binaural Hearing

While the monaural ABR provides information about binaural areas of the brain stem (i.e., potentially peaks III and IV), since they are elicited by either sound played directly to one ear (closed field) or equally to both ears (open field), little information can be gained about binaural integration of sound information. We used the BIC of the ABR to measure the ability of the binaural processing of the brain stem as the BIC varies with ITDs played to both ears. We saw no differences in amplitude of the BIC at any ITD between the two genotypes (*p* = 0.809) or with sex (*p* = 0.6904, Figures 5A,B), although there was a significant difference between Fmr1 male and female mouse BIC amplitudes at 1.5 ms ITD. There were no differences between the sexes for B6 mice for any ITD amplitude. Latency of the BIC was significantly slower in male Fmr1 compared with that in B6 males (Figure 5C, lower panel) only at 0 ITD, with no difference in genotype for female mice (Figure 5C, upper panel). When data were combined for sexes across genotypes, there was no significant difference in the latency of the BIC at any ITD (Figure 5D). There were differences in latency of the BIC between B6 (-1.5 ms) and Fmr1 (1 ms) males and females although there was no overall main effect of sex (*p* = 0.3367).

## Discussion

This is the first study to characterize the ABR in the C57BL/6J Fmr1 mutant mouse and, in particular, highlights morphological characteristics, hearing range, monaural ABRs, and binaural integration across sexes and in heterozygote females. Consistent with previous study, we saw an increase in the hearing threshold at high frequencies in Fmr1 mice, although this phenotype is male specific and no change in morphology (pinna or facial characteristics) (McCullagh et al., 2020a). Female Fmr1 mice have reduced wave IV amplitudes of the monaural ABR, and wild-type females have increased wave I and IV amplitudes compared with B6 males, suggesting that female Fmr1 mice have a more male-like phenotype for monaural ABR amplitude. Finally, we showed that male Fmr1 mice have increased latency of the BIC at 0 ITD but not other ITDs or changes in amplitude of the BIC across ITD compared with B6 animals, suggesting changes in the timing of the processing of binaural information that does not change overall ITD following ability.

The pinnae size and shape are the first two features available to determine sound localization ability in animals with external ears (Butler, 1975; Musicant and Butler, 1984). Craniofacial alterations including prominent ears and elongated face are hallmark features of humans with FXS (Loesch et al., 1988; Heulens et al., 2013) and indeed may be a factor in auditory hypersensitivity that has been underexplored. Consistent with our previous study, we saw no alterations in the pinna or facial characteristics in the C57BL/6J mouse model with FXS (McCullagh et al., 2020a) using calipers as a measurement tool. Others have explored differences in the morphological skull in mice with FXS using different tools, such as CT/MRI (Ellegood et al., 2010) and micro-CT (Heulens et al., 2013) with mixed results. Heulens et al., 2013 showed alterations in skull and jaw characteristics that had not been characterized previously with a similar technique (Ellegood et al., 2010) although differences may be due to how features were measured. We also saw no difference in weight of Fmr1 animals compared with the wild-type, which is in contrast to our previous study where we noted that Fmr1 animals weighed less than wild-type (McCullagh et al., 2020a) and others that showed an increase in male Fmr1 mouse weight compared with the wild-type (Leboucher et al., 2019). Differences in weight may be due to the inclusion of female animals (McCullagh et al., 2020a) and older animals (Leboucher et al., 2019). Overall changes in pinna morphology may still be an important factor in sound localization ability in Fmr1 animals and should be explored with more detailed techniques to determine if increased pinna measures in both humans and animal models may underly some aspect of auditory hypersensitivity symptomology.

Our previous results showed increased hearing thresholds at high frequencies (16 kHz) measured by ABR in the C57BL/6J Fmr1 strain with data combined for the sexes (McCullagh et al., 2020a). In the current study, we do not see increased thresholds at 16 kHz but do see a trend towards increased thresholds at higher frequencies in male Fmr1 mice specifically, though not significant. These data are consistent with the increased thresholds across frequencies seen in adult male FVB Fmr1 mice (Rotschafer et al., 2015), though note that there was no change in threshold across frequencies in males of the same FVB strain at younger ages (Kim et al., 2013; El-Hassar et al., 2019). Additional studies should examine the hearing range across development and sexes in both strains to further show whether loss of high-frequency hearing is a conserved feature in FXS.

Previous studies in the FVB Fmr1 mouse line show a robust wave I amplitude decrease in males across ages (Rotschafer et al., 2015; El-Hassar et al., 2019), although see Kim et al. (2013). We did not see any change in wave I amplitude in the C57BL/6J Fmr1 line in adult animals of either sex. These conflicting results may be in part due to the earlier onset age-related hearing loss, which can be seen as decreases in early waves of the ABR, that occurs in the B6 background (Hunter and Willott, 1987). Changes in wave I amplitude specific to FXS may be masked by overall decreases in wave I amplitude across genotypes in this background. Interestingly, data in male FVB Fmr1 mice show no differences (adults, Kim et al., 2013; Rotschafer et al., 2015) or increased amplitudes in wave IV of the ABR (young, El-Hassar et al., 2019), whereas our data show a decreased wave IV amplitude in Fmr1 females on the B6 background. These differences again may be due to differences in sexes and ages of animals tested. Finally, our finding of no difference in latency of monaural waves is consistent with the majority of the work in FVB mice (Rotschafer et al., 2015; El-Hassar et al., 2019), although note that Kim et al., 2013 showed shorter latency for wave I. Our data further add to the knowledge of ABR phenotypes that might be consistent across genotypes.

While ours is the first study to characterize the BIC in an FXS-mutant mouse strain, our data are consistent with the BIC as it varies with ITD in mice (Benichoux et al., 2018). Namely, mice have a small range of ITD cues available due to their small head size, and therefore, the BIC amplitude decreases with increasing ITD between the ears, but this overall amplitude change is smaller than animals with more dominant ITD hearing ability (such as chinchilla or cats)(Benichoux et al., 2018). Additionally, consistent with previous study, the BIC latency gets longer with increasing ITD (Ferber et al., 2016; Laumen et al., 2016; Benichoux et al., 2018). Interestingly, our work in mice with FXS is consistent with an increased latency of the BIC seen in a study in autistic people (ElMoazen et al., 2019), although they also see a decrease in the amplitude of the BIC. Our findings that the BIC latency is only significant in males at 0 ITD potentially suggest that there is overall slowing of binaural processing in the brain stem, which may ultimately impact binaural hearing, but that it is not dependent on ITD, which would be consistent with mice that do not rely as predominantly on ITD cues compared with other species. In addition, while these results do not directly measure auditory hypersensitivity, underlying alterations to the timing of brain stem or amplitude of brain stem regions will impact later processing of this information as it moves through the ascending auditory pathway to other subcortical and cortical areas.

The subject of sex differences in animal models is important for fully understanding the complexities of disorders such as ASD or FXS, which seem to impact females differently than males (Werling and Geschwind, 2013; Nolan et al., 2017). In FXS, due to it being an X-linked disorder, there is a higher prevalence in males than females, which can undergo X-inactivation on the effected X chromosome (i.e., genetic mosaicism) (Kirchgessner et al., 1995). However, mice offer a unique opportunity to measure both heterozygote and homozygous females giving insight into potential sex differences related to loss of Fmr1 on one or both X chromosomes. Our data suggest that there are indeed differences in auditory phenotypes between heterozygous and homozygous females (wave IV amplitude) in addition to differences between males and females. These and future data comparing female Fmr1 subtypes may give insight into the role of X-inactivation in phenotypes of auditory brain stem processing.

## Conclusion

This study offers important insight into auditory phenotypes that may be shared or differ between background strains of mice with FXS. In addition, while subtle, we showed sex-specific and full or heterozygote mutation-specific differences in the auditory brain stem function for both monaural and binaural hearing in B6 background mice. Further studies measuring auditory phenotypes for B6 mice in earlier ages across the sexes would be useful to further characterize potential similarities compared with the FVB Fmr1 strain. In addition, characterizing the BIC in the FVB strain would be useful to elucidate if latency phenotypes are consistent across backgrounds.

## Data Availability Statement

The raw data supporting the conclusions of this article will be made available by the authors, without undue reservation.

## Ethics Statement

The animal study was reviewed and approved by Oklahoma State University IACUC.

## Author Contributions

EM and AC collected the data for the manuscript. EM performed the statistical analyses, created the figures for the manuscript, and developed the ideas and methods. Both authors helped write and revise the manuscript.

## Conflict of Interest

The authors declare that the research was conducted in the absence of any commercial or financial relationships that could be construed as a potential conflict of interest. The handling editor declared a past co-authorship with one of the authors EM.

## Publisher’s Note

All claims expressed in this article are solely those of the authors and do not necessarily represent those of their affiliated organizations, or those of the publisher, the editors and the reviewers. Any product that may be evaluated in this article, or claim that may be made by its manufacturer, is not guaranteed or endorsed by the publisher.
